# Federal Cuts and Public Health: Social Media Sentiment Among Federal Employees

**DOI:** 10.2196/92199

**Published:** 2026-07-15

**Authors:** Yi Wang, Andrew N Crenshaw, Rodrigo Reis

**Affiliations:** 1 People, Health & Place Unit, Bursky School of Public Health, Washington University in St. Louis, One Brookings Drive, St. Louis, MO, 63130, United States, 1 314-935-5000

**Keywords:** federal workforce, mental health, social media, sentiment analysis, public health workforce

## Abstract

Using sentiment analysis of 44,216 public health–related posts from the FedNews Reddit forum (2020-2025), we found that fear and anger scores rose 32% and 63%, respectively, in 2025 over 2024. These results underscore the adverse mental health impact of federal workforce changes and funding cuts on public health employees.

## Introduction

The rapidly evolving federal priorities from 2025 are reshaping the public health landscape in the United States, which involves extensive restructuring of key health agencies, drastic reductions in federal research funding, abrupt termination of contracts with academic institutions, and a federal government shutdown [1,2]. Although the long-term and short-term effects of these changes are not yet fully understood, their implications have garnered considerable attention from social media. The rise of social media platforms over the past two decades has significantly transformed how individuals access and interpret health information, often amplifying both positive and negative sentiments [3]. Social media also provides a timely and proactive data source for understanding the impact of changing health policies on population well-being [4,5]. This study uses Reddit data to investigate how disruptions in federal public health funding and the federal workforce have influenced public sentiment among federal employees.

## Methods

### FedNews Reddit Submissions

We analyzed 197,874 Reddit posts and comments (hereafter “submissions”) from the FedNews Reddit forum, also known as the r/FedNews subreddit (hereafter “FedNews”), made between January 1, 2020, and December 31, 2025. Submissions were downloaded from the Arctic Shift project. With the rapid downsizing of the US federal government workforce, FedNews has become an increasingly popular platform for employees to anonymously share advice with colleagues and voice grievances about the Trump Administration [6]. This makes FedNews a valuable source for understanding employee sentiment. To focus on public health–related discussions, submissions were filtered using search terms representing key US health agencies and programs (eg, Centers for Disease Control and Prevention [CDC], Medicaid; full list in Table A1 in Multimedia Appendix 1). These terms were selected to capture submissions discussing key federal health agencies, programs, and policies, ensuring comprehensive coverage of health-related discourse.

### Sentiment Analysis and Topic Modeling

We used sentiment analysis to measure expressions of fear and anger. Submissions were tokenized, stop words with little or no semantic meaning were removed, and words were stemmed to their root form using the Natural Language Toolkit library in Python. Word-Emotion Association Lexicon was used to annotate text with emotions [7] (Text A1 in Multimedia Appendix 1). We then calculated fear and anger scores for each submission, with values ranging from 0 (lowest, no words denoting fear/anger emotion) to 1 (highest, all words denoting fear/anger emotion). We trained a latent Dirichlet allocation model [8] using submissions across all years, assigned each post a dominant topic, and then plotted how topic share shifted month by month.

### Ethical Considerations

This study obtained approval from the institutional review board at the authors’ home institution (202504154).

## Results

**Figure 1.**
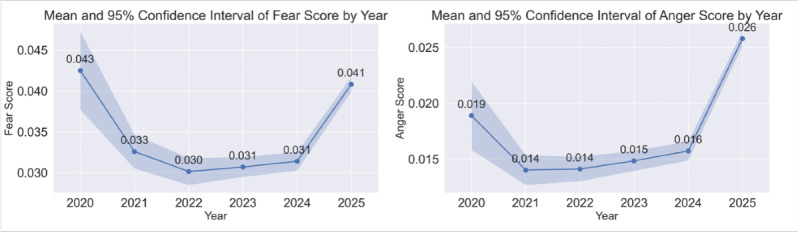
Mean fear and anger scores of public health–related submissions posted to the FedNews Reddit forum, 2020‐2025.

We identified 44,216 FedNews submissions related to public health between January 1, 2020, and December 31, 2025. The volume of public health–related submissions grew significantly over this period, increasing from 519 in 2020 to 1794 in 2021, 2880 in 2022, 7511 in 2023, 10,605 in 2024, and 20,907 in 2025 (Table A2 in Multimedia Appendix 1). The sentiment analysis showed significant increases in expressions of fear and anger in public health–related submissions in 2025 compared to 2021‐2024 (Figure 1).

The average fear score for health-related submissions increased from 0.031 in 2024 to 0.041 in 2025, a substantial 32% increase. The average fear score in 2025 had nearly equaled those seen during the early COVID-19 pandemic in 2020 (see Figure A1 in Multimedia Appendix 1 for month-to-month change). Example submissions with high fear scores include (all submissions quoted have been paraphrased to protect the privacy of the posters/commenters):

*Before widespread telework options were available, how would the government have functioned during a pandemic? Would they have reduced the workforce to essential employees only, or would they have required everyone to continue working despite the circumstances?*

[submitted in May 2022, fear score=0.154]

*What should I do? I have an upcoming surgery and I’m worried about my health insurance coverage. I haven’t been laid off yet, but it’s clear that none of our jobs are secure. I need the surgery for my health, but I’m afraid of losing my insurance before it happens.*

[submitted in February 2025, fear score=0.130]

The average anger score for health-related submissions rose significantly from 0.016 in 2024 to 0.026 in 2025, marking a 63% increase. Notably, the average anger score in 2025 surpassed that observed during the COVID-19 pandemic in 2020 (*P*<.001, 2-tailed test). Example submissions with high anger scores include the following:

*For several months, I was the sole public-facing employee in my office. Early on, I advocated for hazard pay, but the approval process was incredibly cumbersome and required authorization from the highest levels of our agency. It became too emotionally draining for me to continue the effort, so I gave up. Currently, I am at home with what is likely coronavirus, which is both terrible and frightening....*

[submitted in August 2020, anger score=0.133]

*Is anyone else feeling physically ill from this stress? I swear, my anxiety has never been this bad. As a federal employee still required to go to work but unable to perform my job in public health and community outreach, this has been awful. I’m sure many of you are feeling this same sense of hopelessness. Over the past month, my anxiety has been so severe that it’s now causing physical symptoms. Stay strong, everyone!*

[submitted in November 2025, anger score=0.162]

**Figure 2.**
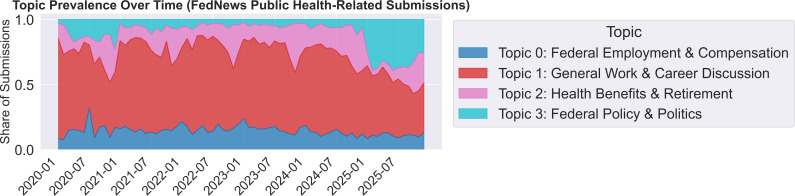
Prevalence of latent Dirichlet allocation–derived discussion topics among public health–related submissions to the FedNews Reddit forum, 2020‐2025.

The topics commonly discussed in public health–related submissions evolved over the years (Table A3 in Multimedia Appendix 1, Figure 2). Prior to 2025, discussions focused on general work arrangements and health benefits. By 2025, the focus had shifted dramatically to issues related to federal politics and policy changes, and their impacts on employees’ health and well-being.

## Discussion

This study found that fear and anger scores in health-related FedNews submissions rose 32% and 63%, respectively, in 2025 compared to 2024—anger surpassing levels seen during the COVID-19 pandemic—while discussion topics shifted from routine work matters to layoffs and policy changes. These findings extend prior survey-based evidence of elevated mental health burden among federal workers [9] and expert warnings about productivity losses from mass layoffs [10].

While the FedNews forum is a rich data source, it represents a self-selected sample of Reddit users who may not be representative of the broader federal workforce. Lexicon-based sentiment analysis, while widely validated in health contexts, cannot capture irony, sarcasm, or domain-specific language nuance. Additionally, while we observe co-occurrence of sentiment shifts and policy changes, causal attribution is not possible from this design. Future research could examine whether these sentiment changes correspond to declines in program performance and physical health outcomes.

A workforce experiencing record levels of fear and anger is less able to perform the high-quality work that underpins food and drug safety, disease surveillance, and emergency preparedness. Policymakers considering further restructuring of federal health agencies should recognize that psychological costs to the workforce are measurable, rapid in onset, and—if left unaddressed—likely to compound the operational disruptions produced by funding cuts alone.

## Supplementary material

10.2196/92199 Multimedia Appendix 1 Supplementary materials on sentiment analysis methodology, search terms, FedNews submission counts, latent Dirichlet allocation topic keywords, and monthly emotion scores.

## Abbreviations

CDC: Centers for Disease Control and Prevention

## References

[1] Galea S (2025). The potential consequences of disinvestment in health in the US. JAMA Health Forum.

[2] Jones C (2025). Government shutdown effects on public health: lessons from the 2025 and 2018-2019 closures. Association of State and Territorial Health Officials.

[3] Chen J, Wang Y (2021). Social media use for health purposes: systematic review. J Med Internet Res.

[4] Valdez D, Ten Thij M, Bathina K, Rutter LA, Bollen J (2020). Social media insights into US mental health during the COVID-19 pandemic: longitudinal analysis of Twitter data. J Med Internet Res.

[5] Hu T, Wang S, Luo W (2021). Revealing public opinion towards COVID-19 vaccines with Twitter data in the United States: spatiotemporal perspective. J Med Internet Res.

[6] Hill K (2025). “Will I lose my job?” Federal workers flock to Reddit for answers. The New York Times.

[7] Mohammad SM, Turney PD (2013). Crowdsourcing a word–emotion association lexicon. Comput Intell.

[8] Jelodar H, Wang Y, Yuan C (2019). Latent Dirichlet allocation (LDA) and topic modeling: models, applications, a survey. Multimed Tools Appl.

[9] Obis A (2025). “A never-ending nightmare”: federal workers detail mental health toll of government downsizing. Federal News Network.

[10] Riddle K (2025). Mental health issues ripple through the federal workforce with firings. NPR.

