# Supplementary material for: Federal Cuts and Public Health: Social Media Sentiment Among Federal Employees
Source: J Med Internet Res. 2026 Jul 15;28:e92199. doi: 10.2196/92199 (PMC13372291; doi:10.2196/92199)
Supplement: Multimedia Appendix 1 [file jmir-v28-e92199-s001.docx]

# Appendix

## Text A1. Lexicon-Based Sentiment Analysis in Health Contexts

The Word-Emotion Association Lexicon (NRC Lexicon), developed by Mohammad and Turney [1], is a seminal resource in the field, mapping thousands of English words to eight discrete emotions (including fear and anger) and two polarity dimensions (positive and negative). Its crowdsourced construction via Amazon Mechanical Turk has made it one of the most widely adopted tools in health-related sentiment studies. Subsequent work has validated its application across diverse health contexts. For example, Boon-Itt and Skunkan (2020) applied NRC-based emotion detection to COVID-19 Twitter data, finding that fear was the dominant emotion throughout early outbreak periods, underscoring the sensitivity of lexicon-based methods to policy-driven emotional responses [2]. Similarly, Xie et al. (2023) used NRC emotion annotation to trace shifting public sentiment in the context of a concurrent natural disaster and public health emergency in China, revealing how the lexicon can capture nuanced temporal dynamics even under compound crisis conditions [3].

References

1. Mohammad SM, Turney PD. Crowdsourcing a Word–Emotion Association Lexicon. Computational Intelligence 2013;29(3):436–465. doi: 10.1111/j.1467-8640.2012.00460.x

2. Boon-Itt S, Skunkan Y. Public Perception of the COVID-19 Pandemic on Twitter: Sentiment Analysis and Topic Modeling Study. JMIR Public Health and Surveillance JMIR Publications Inc., Toronto, Canada; 2020 Nov 11;6(4):e21978. doi: 10.2196/21978

3. Xie Z, Weng W, Pan Y, Du Z, Li X, Duan Y. Public opinion changing patterns under the double-hazard scenario of natural disaster and public health event. Information Processing & Management 2023 May 1;60(3):103287. doi: 10.1016/j.ipm.2023.103287

## Table A1. Public Health Submission Search Terms

| Search Term |
| --- |
| Addiction Services |
| Affordable Care Act |
| CDC |
| Covid |
| Disease |
| Epidemiology |
| FDA |
| Health |
| HHS |
| Medicaid |
| Medicare |
| NIH |
| Nutrition |
| Pandemic |
| Quarantine |
| SAMHSA |
| SNAP |
| Substance Abuse |
| TRICARE |
| Hospital |
| Vaccin* |
| VA |
| VHA |
| WIC |
| Women and Infants |

Note: * is a wildcard representing one or more characters.

## Table A2. Posts and Comments in FedNews by Year.

| Year | FedNews Reddit forum | | | | | |
| --- | --- | --- | --- | --- | --- | --- |
|  | Posts | | | Comments | | |
|  | All | Public-Health-Related | Pct of All Posts | All | Public-Health-Related | Pct of All Comments |
| 2020 | 1,359 | 337 | 25% | 1,265 | 182 | 14% |
| 2021 | 4,303 | 1,184 | 28% | 3,993 | 610 | 15% |
| 2022 | 8,356 | 1,978 | 24% | 6,681 | 902 | 14% |
| 2023 | 14,315 | 3,372 | 24% | 13,325 | 4,139 | 31% |
| 2024 | 18,028 | 4,201 | 23% | 16,455 | 6,404 | 39% |
| 2025 | 63,080 | 11,745 | 19% | 46,714 | 9,162 | 20% |

## Table A3. LDA Topic Keywords among FedNews Public-Health-Related Submissions.

| Topic | Label | Top Keywords (probability weight) |
| --- | --- | --- |
| 0 | Federal Employment & Compensation | pay (.016), leave (.013), opm (.012), agency (.010), employee (.009), gov (.008), may (.007), time (.007) |
| 1 | General Work & Career Discussion | work (.012), job (.010), get (.009), like (.008), people (.007), time (.007), know (.007), one (.006) |
| 2 | Health Benefits & Retirement | health (.024), insurance (.020), plan (.015), healthcare (.010), coverage (.009), retirement (.009), year (.008), care (.008) |
| 3 | Federal Policy & Politics | federal (.013), trump (.009), government (.009), employees (.009), post (.006), administration (.006), department (.004), workers (.004) |


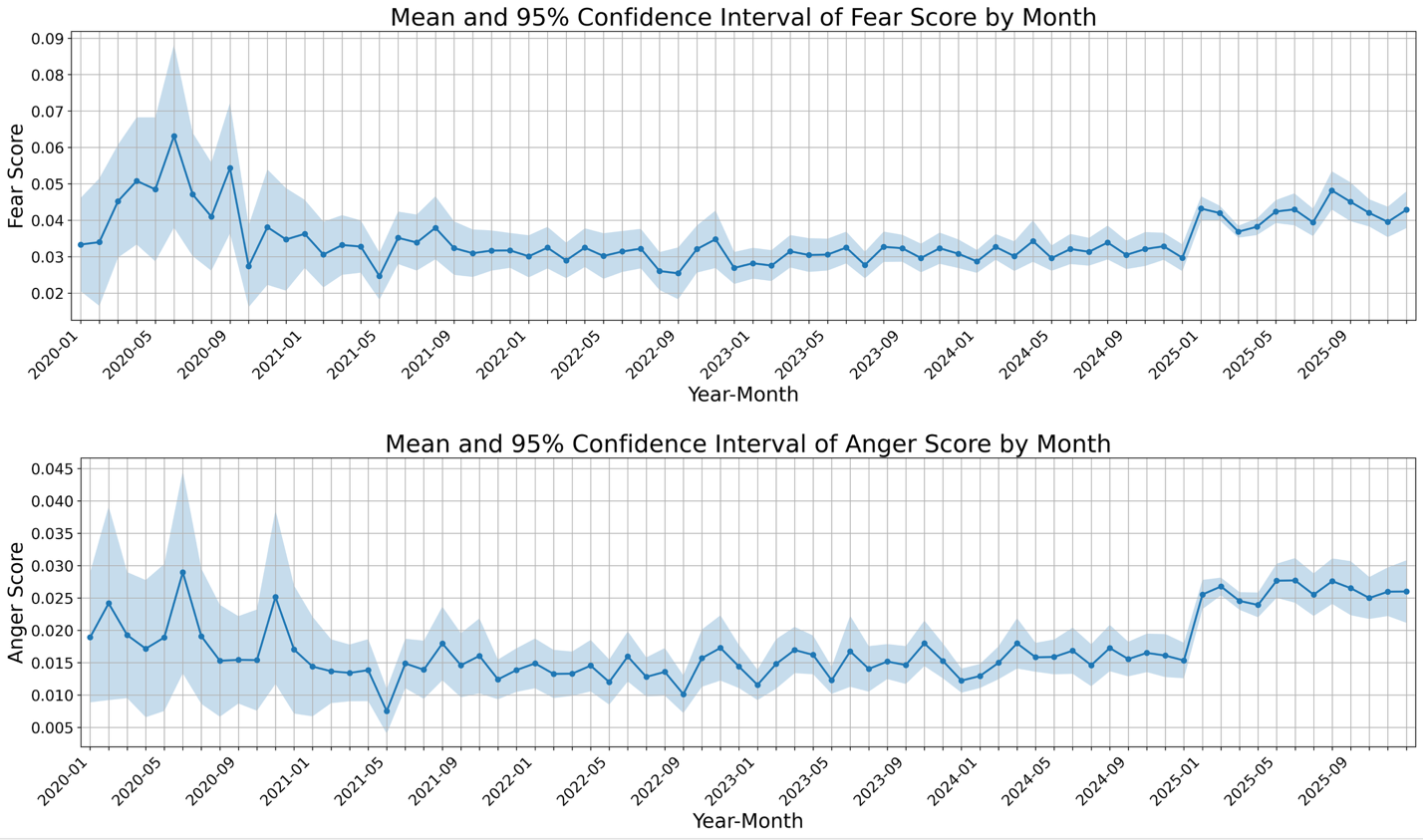


## Figure A1. Fear Score and Anger Score of FedNews Public-Health-Related Submissions by Month.
